# 58-year-old Male with a Headache, Hand Numbness, and Phantosmia

**DOI:** 10.5811/cpcem.2022.4.56508

**Published:** 2022-05-15

**Authors:** Naillid Felipe, Leen Alblaihed, Zachary D.W. Dezman, Laura J. Bontempo

**Affiliations:** * University of Maryland Medical Center, Department of Emergency Medicine, Baltimore, Maryland; † University of Maryland School of Medicine, Department of Emergency Medicine, Baltimore, Maryland; ‡ University of Maryland, Department of Epidemiology and Public Health, Baltimore, Maryland

**Keywords:** status epilepticus, temporal lobe seizure, phantosmia, CPC

## Abstract

**Introduction:**

A 58-year-old male presents to the emergency department with headache, hand numbness, and phantosmia.

**Case Presentation:**

Magnetic resonance imaging showed multiple acute and early subacute lesions involving the cortex and subcortical white matter of the left frontal lobe, left parietal lobe, left temporal lobe, left caudate, and left putamen.

**Discussion:**

This case takes the reader through the subtle findings that led to the diagnosis and ultimately to treatment.

## CASE PRESENTATION (DR. FELIPE)

A 58-year-old Korean male was brought to the emergency department (ED) for evaluation with a chief complaint of two days of acting differently. The patient had no complaints and stated he was unsure why he was in the ED. According to his son, the patient had complained of headaches, bilateral hand numbness, and strange smells for the prior three days. The family noticed the patient was speaking more slowly than usual and replying with one-word answers. The patient had no known past medical or surgical history. He drank four shots of liquor a day and had a 20 pack-year smoking history. He had no recent travel history. A full review of systems was unremarkable.

The patient was alert and oriented to self and date of birth but not to time or place. His temperature was 36.3° Celsius, with a heart rate of 81 beats per minute, respiratory rate of 16 breaths per minute, blood pressure of 142/83 millimeters of mercury, and an oxygen saturation of 96% on room air. He had a body mass index of 28 kilogram per square meter. He appeared well developed and well nourished. His head was normocephalic and atraumatic. He had moist mucous membranes, without oral lesions, and with a normal oropharynx. Pupils were 3 millimeters, equal and reactive to light. The neck was supple and without significant lymphadenopathy, meningismus, or cervical spine tenderness. The patient’s heart beats were regular without murmurs. Breath sounds were clear bilaterally, the abdomen was soft and nontender, and the extremities were warm and well perfused.

Neurologic exam was limited due to the patient’s inability to follow simple commands, but he had grossly intact extraocular movements, symmetric facial movements, grossly intact hearing, and non-slurred speech. His gait was stable, and he ambulated without any assistance. There was grossly 5/5 strength throughout the bilateral upper and lower extremities with normal tone and normal sensation. Speech, given that he was Korean speaking, was difficult to assess, but according to a medical interpreter he was speaking slowly and using neologisms. The skin was warm, dry, and without any rash. The patient was calm, minimally interactive, and staring at the wall. He did not appear to be responding to internal stimuli or hallucinations.

**Image 1:**
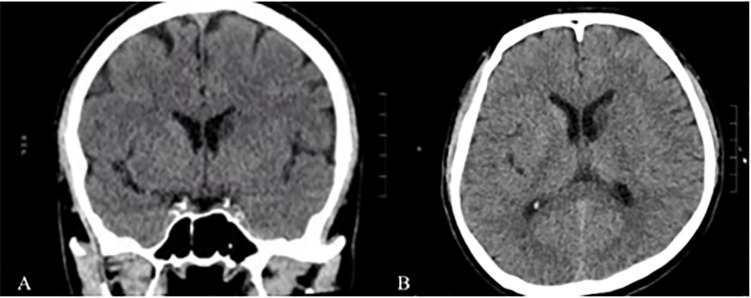
Coronal (A) and axial (B) brain computed tomography representative images of a 58-year-old male presenting with a headache, hand numbness, and phantosmia.

The patient’s initial laboratory test results are listed in Table 1. His electrocardiogram showed normal sinus rhythm, with normal axis, normal intervals and no ST-segment elevation or depression. Due to a concern for possible intracranial bleeding, the patient had a computed tomography (CT) of the head. Representative axial and coronal images are shown in Image 1. Throughout his stay in the ED, the patient remained hemodynamically stable and was able to eat a complete meal. He was noted to be intermittently staring out at the wall and was minimally interactive with staff or his environment. Given his ongoing altered mental status, a lumbar puncture was performed and cerebrospinal fluid (CSF) was obtained. The CSF results are shown in Table 2. A diagnostic test was then done, which confirmed the diagnosis.

## CASE DISCUSSION (DR. ALBLAIHED)

As I was reviewing this case initially, what stood out to me was that this was a clinicopathologic case presentation and, therefore, there had to be an interesting diagnosis behind these vague, subjective symptoms. In the ED, patients who present similarly may be considered for discharge with instructions to follow up with their primary care physician and possibly a psychiatrist if the workup does not reveal a dangerous or treatable cause for the presentation. This patient is a great reminder that new symptoms, as vague and nonspecific they may be, warrant a medical workup before diagnosing, perhaps falsely, a psychiatric etiology, especially when there is no prior history of mental illness. The emergency physician is uniquely situated to catch those patients early, prevent deterioration or death, and improve the quality of their lives.

To recap, the patient’s symptoms include headache, slow speech, olfactory hallucinations, hand numbness, and confusion for three days. This makes the brain the organ that is most likely affected.

On physical examination the patient had normal gait and cranial nerve testing. The most remarkable findings on exam were the noted confusion and difficulty with speech. The blood laboratory tests were non-diagnostic and did not provide an explanation of the patient’s presenting symptoms.

The CSF analysis, however, was abnormal with the presence of marked red blood cells (RBC) and white blood cells (WBC). There was no information about the CSF opening pressure; so it is unknown whether it was normal or elevated. The number of RBCs in the fourth tube is much less (56% less) than that in the first tube. This diminishing, or clearing, of the RBCs is most likely from a traumatic lumbar puncture. In the event of a subarachnoid hemorrhage, I would expect a uniform amount of RBC to be present in all tubes with no clearing.

There was also evidence of xanthochromia on the CSF analysis. Xanthochromia (or yellow pigmentation) is caused by breakdown of RBCs to oxyhemoglobin and then bilirubin. Xanthochromia is determined by two methods: using spectrometry, or visual comparison to a white sheet of paper. In emergency medicine, we are trained to associate the word “xanthochromia” with subarachnoid hemorrhage; however, there are several other reasons it can be present. One explanation would be a traumatic lumbar puncture especially if there were greater than 10,000 RBC present in the first collected tube, as in this case. Other causes of xanthocromia include hyperbilirubinemia and elevated proteins.

**Image 2:**
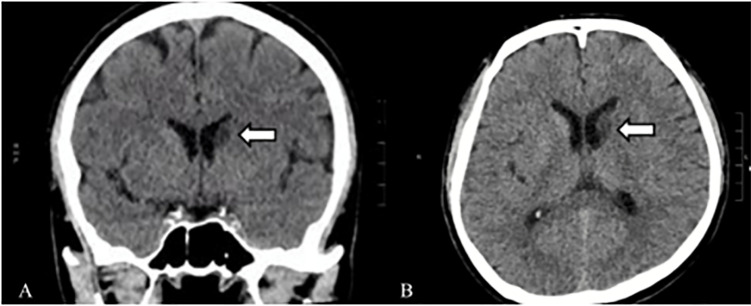
Coronal images of a brain computed tomography without contrast of a 58-year-old male presenting with a headache, hand numbness, and phantosmia showing a hypodensity in the left caudate nucleus in the (A) coronal (arrow) and (B) axial (arrow) views.

When I reviewed the patient’s head CT, I saw a hypodensity in the left caudate nucleus. This can be seen more evidently when you compare both hemispheres in both the axial and coronal views (Image 2). Since the hypodensity is seen in more than one view, it is not artifactual but a real finding. This caudate hypodensity is not as hypodense as the CSF and there is no volume loss (seen as larger ventricle on one side), which means it is not a chronic finding and is more likely acute or subacute, raising concerns for a lacunar infarct or stroke involving the left caudate nucleus.

The caudate nucleus connects the associative cortex (including frontal, parietal, and temporal lobes) with deeper anatomic structures. Symptoms of caudate stroke include the following:

- Cognitive and behavioral changes
- Abulia, which includes decreased spontaneous activity and speech, lack of initiative, indifference, psychic akinesia (affective stagnation), prolonged latency in responding to questions and other stimuli, bradykinesia, and akinetic mutism.

The possibility of a caudate stroke in this patient explains his headache, the CSF findings, the lacunar hypodensity on CT imaging, the confusion or being described as “out of it,” and the lack of speech. However, a caudate stroke does not explain two of the patient’s symptoms: 1) the bilateral hand numbness; and 2) the phantosmia (olfactory hallucinations). The causes of bilateral hand numbness are numerous and include stroke, alcohol use disorder, and paraneoplastic syndrome among others. The causes of olfactory hallucinations includes seizures, intracranial hemorrhage, migraines, Parkinson’s disease, strokes, brain tumors, alcohol withdrawal, and herpes simplex virus (HSV) encephalitis.

The patient has multiple neurological symptoms (olfactory hallucinations, impaired speech, trouble with direction, and apathy/indifference) that point toward involvement of the temporal lobe. However, the caudate nucleus (involved in this case as evident by CT imaging) is not located in the temporal lobe.

Given that the patient is elderly and presenting with confusion, HSV encephalopathy must be on the differential diagnosis list especially in that it can explain his symptoms of olfactory hallucinations as well. The HSV causes a necrotizing infection of the mesocortex and allocortex, usually involving the bilateral temporal lobes, but can involve any part of the brain. The virus is transmitted via the olfactory nerve (causing changes in the sense of smell) or, more commonly, via the trigeminal nerve. Fever is a predominant symptom in HSV encephalitis and is the presenting symptom in 90% of the cases along with progressively worsening mental status changes and confusion. The patient in this case did not have any progression of his confusion over the prior three days. In fact, he was sitting comfortably in the ED eating a sandwich and was afebrile. Other symptoms of HSV encephalitis include headache, psychiatric and personality changes, seizures (focal or generalized), dysphasia, and focal weakness.

If I were clinically taking care of this patient, I would empirically treat him for the possibility of HSV encephalitis pending the CSF culture and polymerase chain reaction result. Herpes simplex virus encephalitis has different presenting symptoms and should be considered in an elderly person with confusion, fever, or when RBCs are found in the CSF. I would also obtain brain and cervical spine magnetic resonance imaging (MRI) and I would place the patient on continuous electroencephalogram (EEG) monitoring to assess for subclinical seizures, focal seizures or status epilepticus. All of which could be caused by infections or cerebral ischemic changes, as evident in the patient’s non-contrast CT showing the presence of caudate hypodensity, possibly indicating an infarct.

My main differential diagnoses in this case are as follows:

- HSV encephalitis:
  ○ This would explain his personality change, dysphasia, headache, olfactory hallucinations (either through direct invasion of olfactory nerve, or through temporal lobe damage resulting in seizures).
  ○ This would not explain the lack of fever and not having progressive worsening of his condition.
- Caudate stroke
  ○ This would explain the CT finding, the CSF finding, his behavioral and cognitive changes.
  ○ This would not explain the olfactory hallucinations.
- Seizures (mesotemporal)
  ○ This would explain his change in behavior and cognition, olfactory hallucinations, trouble following direction, and speech defect. However, seizure symptoms are usually intermittent as opposed to being continuous and unchanged for three days.
  ○ This would not explain the caudate hypodensity presented on the CT imaging and lack of temporal lobe involvement on the CT.

Given the patient’s overall presentation and ED workup, my test of choice for this patient would be an MRI to diagnose a stroke to the caudate nucleus and temporal lobe, with possible seizures as a result. This patient will also need a continuous EEG since the possibility of ongoing seizures remains.

## CASE OUTCOME (DR. FELIPE)

The diagnostic study used in this case was a continuous EEG which was initiated in the ED. The patient was admitted to the neurology service. The EEG showed multiple, prolonged, left mid-temporal electrographic seizures consistent with nonconvulsive status epilepticus (NCSE) originating in the temporal lobe. An MRI study was obtained, which showed multiple acute and early subacute lesions involving the cortex and subcortical white matter of the left frontal lobe, left parietal lobe, left temporal lobe, left caudate, and left putamen, thought to be thromboembolic in origin.

While hospitalized, the patient was started on the anti-epileptic medications fosphenytoin and levetiracetam. Given concern for the temporal lobe as the focus of seizures and hemorrhagic lumbar puncture results, the patient was started on acyclovir to empirically treat for HSV encephalitis; however, following negative viral studies, treatment was discontinued. Additionally, the patient had a bilateral carotid ultrasound, which showed significant carotid stenosis of the left common carotid artery and internal carotid artery. His mental status returned to baseline. He continued on anti-epileptic medications, was advised to cease tobacco and ethanol use, and was discharged home.

## RESIDENT DISCUSSION (DR. FELIPE)

Status epilepticus arises when mechanisms for seizure termination falter, resulting in prolonged epileptiform activity with risk of long-term consequences.^1^ Status epilepticus is defined as seizure activity lasting more than five minutes or recurrent seizures with no return to the patient’s baseline mental status. It can be divided into convulsive and nonconvulsive types.^2^ In NCSE, patients have absence of typical tonic-clonic motor symptoms.^1^ Nonconvulsive status epilepticus is defined as a persistent change in mental status without motor symptoms but with evidence of seizure activity on EEG.^2^ There are two subtypes of NCSE: generalized NCSE, and focal NCSE.^3^

The incidence of NCSE is difficult to report due to the varying definitions throughout the years and overall difficulty in diagnosing the condition. Up to 50% of all cases of status epilepticus might be NCSE. 4 The mortality and morbidity of NCSE is predicted to be as high as 65%, although studies have shown inconsistent results. 5

Clinicians should have a high index of suspicion for NCSE in patients with a history of a seizure disorder, recent changes in anti-epileptic medications, prolonged postictal state after a generalized tonic-clonic seizure, or unexplained altered mental status.^4^ The signs and symptoms of NCSE will vary depending on the subtype of NCSE with which the patient presents. Generalized NCSE usually presents as an absence seizure, which is characterized by perioral and eyelid myoclonus.^3^ Focal NCSE will differ depending on the lobe affected.^3^ A frontal lobe NCSE affects cognitive functions. Temporal lobe NCSE affects autonomic functions. Parietal lobe NCSE affects somatosensory functions, and occipital lobe NCSE affects visual function.^3^ The symptoms could be further divided into negative and positive symptoms.^5^ Negative symptoms include anorexia, confusion, aphasia, amnesia, and/or staring.^5^ Positive symptoms include delusions, blinking, automatisms, lip smacking, facial twitching, perseveration, nausea/vomiting, laughter, and/or crying. 5

As noted above, NCSE can present with many different signs and symptoms. The workup in the ED will be geared toward the patient’s presentation and the severity of illness at the time of evaluation. The etiology of NCSE can be broad, encompassing metabolic derangements, toxicology, infections, trauma, encephalopathy, and primary neurological disease among others. Nonetheless, it is essential to keep NCSE in the differential diagnosis and to consider NCSE in patients who are postictal for an extended period.^4^ It is recommended to start with labs including a complete blood count, comprehensive metabolic panel, urine analysis, and also obtain an electrocardiogram and CT of the brain. If these are inconclusive, consider a lumbar puncture and neurology consultation for urgent EEG initiation.^2^,^4^ It is paramount to consider diagnoses that can mimic NCSE such as psychiatric disturbances, migraine auras, strokes, transient ischemic attacks, and transient global amnesia.^6^

In patients with suspected NCSE the first line of treatment is benzodiazepines, such as lorazepam at 0.1 milligrams per kilogram intravenously.^4^,^5^ Of note, a clinical improvement immediately after antiepileptic medication is given is not sufficient evidence to rule out NCSE.^5^ An EEG should be used to determine when to stop treatment of rapid-acting antiepileptic drugs.^5^

## FINAL DIAGNOSIS

Left temporal lobe nonconvulsive status epilepticus secondary to ischemic stroke.

## KEY TEACHING POINTS

- Consider nonconvulsive status epilepticus in patients with altered mental status and a prolonged postictal state after the visible seizure activity has stopped.
- Temporal lobe epilepsy can present with auras and automatisms.
- Nonconvulsive status epilepticus is treated initially with benzodiazepines. Continuous EEG monitoring can be used to decide when to stop treatment. Long-term treatment is with anti-epileptic medications.

## Figures and Tables

**Table 1:** Blood laboratory results of a 58-year-old male presenting with a headache, hand numbness, and phantosmia.

Test Name	Patient Value	Reference Range
Complete Blood Count		
White Blood Cell	6.6 K/mcL	4.5 – 11 K/mcL
Hemoglobin	13.2 g/dL	11.9 – 15.7 g/dL
Hematocrit	39.8%	35.0 – 45.0%
Platelets	240 K/mcL	153 – 367 K/mcL
Complete Metabolic Panel		
Sodium	135 mmol/L	136 – 145 mmol/L
Potassium	4.2 mmol/L	3.5 – 5.1 mmol/L
Chloride	104 mmol/L	98 – 107 mmol/L
Bicarbonate	23 mmol/L	21 –30 mmol/L
Blood urea nitrogen	18 mg/dL	7 – 17 mg/dL
Creatinine	0.77 mg/dL	0.52 – 1.04 mg/dL
Glucose	107mg/dL	70–100 mg/dL
Albumin	4.1 g/dL	3.2 – 4.6 g/dL
Total bilirubin	0.8 mg/dL	0.3 – 1.2 mg/dL
Aspartate aminotranferase	23 units/L	14 – 36 units/L
Alanine aminotransferase	10 units/L	0 – 34 units/L
Alkaline phosphatase	67 units/L	38 – 126 units/L
Additional Labs		
Acetaminophen	<10.0 mcg/mL	<10.0 mcg/mL
Salicylate Level	<1.0 mg/dL	<1.0 mg/dL
Ethanol Level	<10.0 mg/dL	<10.0 mg/dL

*K*, thousands; *mcL*, microliter; *g*, grams; *dL*, deciliter; *mmol*, millimole; *L*, liter; *mg*, milligram; *mcg*, microgram; *mL*, milliliter.

**Table 2:** Cerebral spinal fluid results of a 58-year-old male presenting with a headache, hand numbness, and phantosmia. Tube 1 is the first tube obtained during lumbar puncture; Tube 4 is the last tube obtained during lumbar puncture.

Test	Tube 1	Tube 4	Reference Range
Glucose	62 mg/dL		40–85 mg/dL
Protein	608 mg/dL		15–45 mg/dL
WBC	55 cells/mm^3^	22 cells/mm^3^	0 to 5 cells/mm^3^
Polycytes	30%	42%	0–5%
Lymphocytes	70%	50%	40–80%
Monocytes	N/A	4%	15–45%
Eosinophils	N/A	4%	0–10%
RBC	402,500 cells/mm^3^	177,500 cells/mm^3^	0 cells/mm^3^
CSF pre-centrifuge color	Bloody	Bloody	Colorless
CSF pre-centrifuge clarity	Cloudy	Clear	Clear
CSF post-centrifuge color	Xanthochromic	Xanthochromic	Colorless

*dL*, deciliter; *mg*, milligram; *mm^3^*, cubic millimeter; *WBC*, white blood cells; *RBC*, red blood cells, *CSF*, cerebrospinal fluid.
